# Early Detection of Temperament Risk Factors: A Comparison of Clinically Referred and General Population Children

**DOI:** 10.3389/fpsyt.2021.667503

**Published:** 2021-06-24

**Authors:** Marcel Zentner, Vivienne Biedermann, Christina Taferner, Hannah da Cudan, Eva Möhler, Hannah Strauß, Kathrin Sevecke

**Affiliations:** ^1^Department of Psychology, University of Innsbruck, Innsbruck, Austria; ^2^Department of Child and Adolescent Psychiatry, Medical University of Innsbruck, Innsbruck, Austria; ^3^University of Heidelberg, Universitätsklinik des Saarlandes, Homburg, Germany

**Keywords:** preschool, child temperament, behavior problems, assessment, screening, clinically referred, externalizing behavior problems

## Abstract

Despite an extensive literature on associations between early childhood temperament and behavior problems, most of this evidence is based on general population samples. Hence, relatively little is known about the temperament characteristics of children who have been referred for in- or outpatient treatment of emotional and/or behavioral problems. Whether temperament-to-behavior problems identified in community samples would also be found in samples of clinically referred children is poorly understood. To redress this limitation, we compared temperament attributes of a predominantly preschool-aged sample of children referred for treatment of emotional and/or behavioral disorders (*N* = 87) with those from a similarly-aged general population sample (*N* = 85) by using the Integrative Child Temperament Screener (ICTS)—a new nine-item scale to identify clinically significant temperament attributes. Behavioral symptoms in the clinical sample were assessed through diagnostic interviews in combination with the Child Behavior Checklist (CBCL), which was also administered to the general population children. Compared with general population children, referred children exhibited substantially higher scores on all ICTS subscales except behavioral inhibition. Furthermore, areas under the curve analyses showed that discrimination of both groups based on CBCL scales could be improved by adding the ICTS. Overall, the findings fill a long-standing gap in evidence regarding temperament characteristics of children with serious emotional and/or behavioral symptoms and suggest a useful role for the ICTS in assessment, screening, and prevention.

## Statement of Public Significance

This study provides one of the first demonstrations that temperament risk factors identified in general population studies are also exhibited by children referred for psychiatric treatment, albeit in more marked form. Furthermore, the findings indicate that these predisposing temperament factors can be accurately screened for by using a nine-item scale.

## Introduction

Early childhood temperament is one of the few behavioral characteristics that has been found to predict clinically significant outcomes beyond childhood, sometimes up to adulthood (1). In general, studies suggest that certain attributes of preschool temperament shape risk for anxiety, depression, attention-deficit hyperactivity, and conduct problems (2–5). Most of the evidence for associations between early child temperament and later psychopathology so far, however, derives from general population studies. Less is known about the temperament attributes of preschool children who have been clinically referred for in- or outpatient treatment of emotional and/or behavioral problems (6). One reason for the limited number of studies of preschool temperament in clinically referred children may lie in the practical difficulties involved in studying special populations; another is the considerable length of well-established temperament questionnaires such as the Children's Behavior Questionnaire or the Junior Temperament and Character Inventory, which may overtax the temporal or attentional resources available in special population settings.

Examining temperament attributes in clinically referred preschool children is important for at least four reasons. First, a comparison of clinically referred children with general population children can provide insights as to whether the temperament attributes involved in mild behavior problems differ from those involved in more severe problems and, if so, whether they differ in magnitude or in kind. Second, young children referred for psychiatric treatment are those who need the most support and who would particularly benefit from an understanding of predisposing temperamental factors. Third, if temperament risk factors can be discerned early, when brain and behavioral plasticity is relatively high, this gives interventions a better chance to succeed. Fourth, the merits of temperament scales in detecting early appearing temperament risk factors remain limited as long as their capacity to discriminate normally developing children from children referred for social and emotional behavior disorders remains unknown.

Researchers interested in measuring temperament in children referred for mental health care face a large number of child temperament measures that often include age-specific variants for the infancy, toddler, preschool, and school periods (7). Such diversity can prove confusing and make results across instruments difficult to compare. However, if the interest of the researcher lies primarily in capturing key vulnerability factors for the development of behavior problems over the long term, it is possible to significantly reduce the vast number of traits and scales to a few characteristics that are represented across most models of temperament.

Indeed, most of the evidence for persistent, long-lasting effects of early childhood temperament crystallizes around three temperament components. Behavioral inhibition, which is related to harm avoidance, is a well-documented risk factor for the development of later anxiety and depressive symptoms [e.g., (8, 9)]. Anger/frustration, as well as low effortful control, have been found to predict various types of externalizing problems, including attention deficit hyperactivity disorder, substance dependence, and conduct and antisocial personality disorders [e.g., (10–13)]. The latter two dimensions often compound one another in putting children at risk for externalizing behavior problems. Thus, toddler inattention and impaired emotion regulation, as measured in response to a frustration task, were found to be powerful predictors of a chronic externalizing profile (14). They also coalesce in the construct of undercontrol—a clinically significant cluster defined by traits such as impulsivity, inattention, and emotional volatility (15). A similar cluster characterized by low attentional focusing, low approach tendencies, anger/frustration, and impulsivity was found to predict poor adaptive functioning in children with autism (16). It is also noteworthy that the combination of negative emotionality, notably anger, and low effortful control was more strongly associated with violent and non-violent delinquency than psychopathic traits and childhood traumatic events in a sample of juvenile offenders (5). Long-term behavior problems associated with these early childhood temperament attributes are summarized in Tables 1A,B.

**TABLE 1A:** Infant-to-preschool temperament predictors of adolescent and adult personality and psychopathology: undercontrol/inattention.

**Longitudinal** **study**	**Early childhood** **temperament**	**Adolescent/adult** **outcomes**	**Predictive** **range**
Dunedin Health and Development Study	Undercontrol/impulsivity	Elevated suicide risk Criminal offendingSubstance dependence	3–18 years 3–26 years 3–32 years
Mauritius Child Health Project	Fearlessness, disinhibition	Psychopathy	3–28 years
Block & Block Longitudinal Project	Ego-undercontrol	Ego-undercontrol Narcissism	3–23 years 3–23 years
Colorado Longitudinal Twin Study	Impulse control	Executive functions	18–36 months to 16–17 years
Mannheim Longitudinal Study	Attentional deficits	Novelty seeking	3 months to 16 years
Fullerton Longitudinal Study	Temperamental difficulty	Externalizing and internalizing behaviors	18 months to 17 years

*Tables 1A and 1B adapted from Zentner (1, 17)*.

**TABLE 1B:** Infant-to-preschool temperamental predictors of adolescent and adult personality and psychopathology: inhibition/fearfulness.

**Longitudinal** **study**	**Early childhood** **temperament**	**Adolescent/adult** **outcomes**	**Predictive** **range**
Harvard Longitudinal Study	High reactivity	Trait anxiety Amygdala hyperresponsiveness	4 months to 15 years 4 months to 21 years
University of Maryland Longitudinal Study	Inhibition	Internalizing problems	14 months to 26 years
Dunedin Health and Development Study	Inhibition	Depression Harm avoidance	3–18 years 3–26 years
LOGIC Study	Inhibition	Internalizing problems	4–23 years
Uppsala Longitudinal Study	Shyness	Social anxiety Depressive symptoms	20 months to 21 years 20 months to 21 years
Bernese Longitudinal Study	Infant reactivity Irritability	Shyness	3–4 months to 15 years

A tool for measuring these attributes, the Integrative Child Temperament Screener (ICTS), was recently introduced (17). Derived from the 30-item Integrative Child Temperament Inventory [ICTI (18)], it assesses anger/frustration, behavioral inhibition, and attentional persistence as key temperamental vulnerability factors that are represented across models of child temperament (hence the instrument's designation as “integrative”). Because anger/frustration is a risk factor for developing externalizing problems especially when it co-occurs with low attentional persistence, the instrument also makes provision for computing a score that combines both scales. We termed this composite dimension impulsivity, since children with low frustration tolerance and difficulties focusing attention often appear to behave impulsively. However, we realize that terms such as “undercontrol,” “low self-control” or “temperamental difficulty” could have been used instead, and that impulsivity has a more circumscribed meaning in certain temperament theories and inventories. Table 2 provides a summary of the characteristics measured by the ICTS, along with related temperament dimensions.

**TABLE 2:** Summary and capsule definitions of temperament dimensions included in the ICTS.

**ICTS dimension** **(number of items)**	**Capsule definitions**	**Examples of related dimensions**
Behavioral inhibition (3)	Inhibition of behavior in response to novel unfamiliar people and situations	Harm avoidance (JTCI)^a^; shyness (CBQ, EAS); social fearfulness (TBAQ)
Anger/frustration (3)	Aggressive or irritated behavior in response to painful and/or frustrating input	Anger/frustration (CBQ); anger (TBAQ); distress to limitations (ICQ)
Attentional persistence (3)	Capacity for attentional focusing and control as the basis for voluntary behavior, including persistence	Effortful control (CBQ), persistence (JTCI); interest (TBAQ); distractibility (BSQ)
Impulsivity^b^	Low tolerance for frustration combined with low self-regulatory abilities	Negative emotionality (CBQ), difficultness (ICQ), undercontrol (15)

a *Initials refer to questionnaires that include the listed scales. BSQ, Behavioral Style Questionnaire (19); CBQ, Child Behavior Questionnaire (20); EAS, EAS Temperament Survey for Children (21); ICQ, Infant Characteristics Questionnaire (22); JTCI Junior Temperament and Character Inventory (23); TBAQ, Toddler Behavior Assessment Questionnaire (24)*.

b *Composite variable composed of anger/frustration and attentional persistence*.

An important consideration in designing the ICTS was that it should measure the vulnerability factors shown in Table 2 in an economical, developmentally appropriate, and cross-nationally comparable way. Thus, each ICTS attribute is measured with three items only, resulting in a nine-item scale that can be administered in about 1 min (17). Items representing each component were specifically selected on the basis of their developmental suitability and measurement invariance across the period from 2 to 8 years of age. For example, attentional persistence was included as the facet of effortful control with the greatest likelihood of exhibiting measurement invariance from infancy to school age. The items have also shown measurement invariance across several nations (17). Demonstration of measurement invariance is important because it ensures that items retain the same meaning across different age or national groups, thus allowing for unbiased cross-temporal or cross-national comparisons.

As would be expected from previous research, in a community sample of preschoolers, ICTS anger/frustration has been found to be distinctively associated with mother-reported conduct problems, lack of attentional persistence with hyperactivity symptoms, and inhibition with emotional symptoms (17). Interestingly, the specific ICTS scales explained considerably more variance in problem behaviors than did broad, higher order factors such as effortful control or negative emotionality (25). However, as is the case for the vast majority of studies, these temperament-to-behavior problem associations were found in general population samples. What little is known about such associations in referred children seems to point to a similar pattern of temperament-to-behavior problem associations (6). However, the scant evidence leaves several questions unanswered. For example, whether the pattern generalizes across nations and assessment instruments and, most notably, which tools may offer sufficient utility to be used in child mental health settings for the identification of an at-risk temperament profile.

To fill this gap, in the current study, we examined temperament characteristics in young children referred for treatment of emotional and/or behavioral disorders and compared them with those from an age- and gender-matched general population sample by using the ICTS. We reasoned that, since the ICTS scales were specifically designed to identify temperament characteristics associated with risk for behavioral problems, the scales should differentiate referred from non-referred children by standard discrimination metrics, such as area under the receiver operating characteristic (AUROC) curve analyses. We were also interested in examining whether the ICTS would add to the prediction of diagnostic status above and beyond the Child Behavior Checklist (CBCL) scales. Finally, to see whether the ICTS could predict, in addition to binary diagnostic status, specific symptom profiles, we formed subgroups that exhibited an internalizing and externalizing symptom profile and repeated the AUROC analyses in relation to these more specific symptom groups.

## Method

### Participants

The clinically referred sample comprised all in- and outpatients treated at the Department of Child and Adolescent Psychiatry and Psychotherapy in Hall in Tirol, Austria between August 2017 and January 2020, for whom temperament ratings were available. This was the case for 87 children aged 2–11 years (*M* = 4.96, *SD* = 1.86), 67.8% of whom were boys. The vast majority of children (87%) were preschoolers (aged 6.5 years or less). This sample was compared with a general population sample assessed as part of a longitudinal study on temperament and development conducted at the University of Heidelberg, comprising 85 children aged 5–6 years (*M* = 5.02, SD = 0.15), 54.1% of whom were boys. Sample characteristics are displayed in Table 3.^1^

**TABLE 3:** Descriptive statistics of clinically referred and general population children.

	**Clinical sample** **(Hall)**	**Population sample** **(Heidelberg)**	***p***
	***N = 87***	***N = 85***	
Clinical treatment			
Inpatient	32 (36.8%)	NA	
Outpatient	55 (63.2%)	NA	
Sex			0.092
Male	59 (67.8%)	46 (54.1%)	
Female	28 (32.2%)	39 (45.9%)	
Age			0.003
(at time of assessment or initial admission)	4.92 (1.81)	5.51 (0.08)	
Relationship status of biological parents			<0.001
Living together	36 (62.1%)	85 (100%)	
Separated/Divorced	20 (34.5%)	0 (0.00%)	
Separated by death	1 (1.72%)	0 (0.00%)	
Never lived together	1 (1.72%)	0 (0.00%)	

To determine the adequacy of the sample size, we conducted a power analysis by using G^*^Power 3.1 (26) for *t*-tests on two independent groups, and for a multiple regression with 10 predictors. The expected effect size was based on a previous study using the ICTS (17), in which problem behaviors correlated with the relevant ICTS dimensions between *r* = 0.43 and *r* = 0.46, averaging *r* = 0.44 (*d* = 0.98; AUC = 0.76). From these criteria G^*^Power estimated a minimum sample size of 72 participants to achieve a power of 1–β = 0.95 and α = 0.05.

### Measures

#### Child Temperament

The ICTI is a 30-item measure that assesses the temperament dimensions of anger/frustration, behavioral inhibition, attention/persistence, activity level, and sensory sensitivity in preschool and early school-age children (18). The nine items of the ICTS are embedded in the ICTI and capture the three clinically most significant scales of the ICTI with three items each (17): anger/frustration (e.g., “cries or yells when asked to stop favorite occupation”); behavioral inhibition (e.g., “is shy when meeting unfamiliar children”); and attentional persistence (e.g., “when looking at a book or painting, is quickly bored and changes activity”). For the sake of brevity, the ICTS dimensions are sometimes simply referred to as frustration (for anger/frustration), inhibition (for behavioral inhibition), and attention (for attentional persistence). A composite trait termed “impulsivity” is defined by low frustration tolerance in combination with poor attentional control. It is computed by adding the anger/frustration and the (reverse-scored) attentional persistence scale items (17). The items are presented on a six-point scale ranging from 1 (*behavior occurs never or hardly ever*) to 6 (*behavior occurs always or close to always*). The complete scale can be found in (7).

#### Child Problem Behavior

Upon admission, children were assessed with a routine diagnostic battery comprising expert ratings and parental reports. Standardized clinical interviews provided essential information for diagnostic classification. Although children aged 6 years and older were assessed with the Kinder-DIPS—a diagnostic interview for assessing mental disorders in children and adolescents (27)—there are no generally accepted, standardized measures for assessing preschool mental disorders in German-speaking countries. Thus, diagnoses for children aged 5 years and younger were primarily based on developmental and disorder-specific measures, as well as behavioral observations.

Clinical diagnoses for all children were determined according to the Multiaxial System (MAS) of the 10th revision of the *International Classification of Diseases* (ICD-10) in combination with criteria from the DC: 0–5 in multidisciplinary classification meetings, in which child and adolescent psychiatrists, clinical psychologists, and psychotherapists evaluated children's symptoms based on anamnestic information, behavioral observations, and questionnaire data. An overview of the diagnoses can be found in Supplementary Table 1. A total of 20 children did not meet the criteria of a specific axis 1-MAS diagnosis but were non etheless referred for treatment at the clinic because of elevated strain. Of the other behavioral and emotional disorders (F98), the majority of children (*n* = 32, 76.2%) were diagnosed with unspecified behavioral or emotional disorders (F98.9), five (11.9%) with non-organic encopresis (F98.1), four (9.5%) with other specified behavioral and emotional disorders (F98.8) and four with eating disorder (F98.2), and two (4.8%) with non-organic enuresis (F98.0). This distribution of behavior disorders is broadly reflective of the prevalence of preschool behavior disorders as identified in large-scale epidemiological studies [e.g., (28–30)]. According to these, the majority of problems fall into the externalizing class (DSM-VI: ADD, ODD, CD; ICD-10: F90-98); followed by internalizing problems (DSM-VI: Depression, SAD, GAD, social phobia; ICD-10: F40-48), and disorders of psychological development (ICD-10: F8).

To assess internalizing and externalizing problem behavior, we asked the caregivers of the clinically referred children to complete the CBCL for ages 1.5–5 (31) or for ages 6–18 (32), depending on the child's age. Caregivers of the children recruited in the population study completed the CBCL for ages 4–18 (33), since this study was conducted before the new CBCL versions became available. Caregivers were asked to rate items on a three-point scale (0 = not at all true, 1 = somewhat true, 2 = very true). Scores for internalizing and total problems were computed in accordance with scoring instructions described in the respective manuals (31–33). In the case of the externalizing scale, we proceeded by following the manual instructions for the CBCL/1.5–5, whereas we added the attention problems subscale to the externalizing composite for CBCL/4–18 and CBCL/6–18. This was done to ensure comparability with the results obtained in an earlier study with the Strengths and Difficulties Questionnaire (17), which includes hyperactivity in the externalizing broadband symptom scale. To make the scores of the different CBCL versions comparable, we computed *T*-scores for the subscales and the broadband syndrome scales according to gender-specific norms reported in the respective manuals. Internal consistency reliabilities for internalizing and externalizing scales of the CBCL ranged from α = 0.89 to α = 0.93. From continuous scores, children can be allocated to normal, borderline, and clinical ranges regarding externalizing, internalizing, and total problems in reference to the respective manuals (31–33). Allocation to the corresponding categorical CBCL risk groups was used for certain types of analyses.

To ensure that parental CBCL ratings were reflective of symptoms as identified in the clinical diagnoses, we compared parental CBCL ratings to the ICD-10 diagnoses and symptoms. The comparisons were carried out on externalizing symptoms because of the ICTS' particular relevance to this symptom class. Agreement was in the moderate range, κ = 0.562 (95% confidence interval 0.384 to 0.740; *p* < 0.001; *n* = 83), for dichotomous ratings (0 = no externalizing symptoms, 1 = externalizing symptoms).

### Procedure

Parents completed the ICTI and the CBCL. The questionnaires in the referred group were mostly completed by the mothers, who were the primary caregivers, and partially by the fathers. For three children of the clinical sample (3.45%), no mother ratings on the CBCL were available and so they were substituted with father ratings. For four children (4.60%), no CBCL ratings were available at all. For eight children (9.20%), no mother ICTI ratings were available, but only father ratings; therefore, the latter were used. In the general population sample, ratings were available by fathers and mothers for the ICTI and by mothers only for the CBCL. To make the ratings comparable with the sample of referred children, we used only maternal ICTI ratings, except for four cases in which missing mother ratings were substituted with the father ratings. For one child (1.18%), neither mother nor father ratings of the ICTI were available. CBCL ratings in the general population sample were available for all but one child (1.18%). Caregivers of the clinically referred children signed informed consents for using data for scientific research and knew that participation in the study would not influence treatment. The study involving the clinically referred group was approved by the medical ethical committee of Innsbruck Medical University.

## Results

### Temperament Traits in Clinically Referred Children Compared With General Population Children

Means, standard deviations, and scale intercorrelations of the combined samples are shown in Table 4. As can be seen, all CBCL subscales and all ICTS scales except inhibition were significantly associated with the clinical status of children. The three significant ICTS-to-diagnostic status correlations were comparable to the CBCL-to-diagnostic status correlations in magnitude, which is somewhat surprising given that the ICTS scales were not designed to directly assess problem behaviors. The ICTS and CBCL scales were plausibly intercorrelated. For example, the highest correlation of ICTS Frustration was with CBCL Aggressive Behavior (*r* = 0.64, *p* < 0.001), the highest correlation of ICTS Inhibition was with CBCL Withdrawn (*r* = 0.45, *p* < 0.001), and the highest negative correlation of ICTS Attention was with CBCL Attention Problems (*r* = −0.67, *p* < 0.001). In line with previously reported findings (17), all ICTS scales exhibited satisfactory internal consistency reliability (Frustration: α = 0.75; Inhibition: α = 0.73; Attention: α = 0.80; Impulsivity: α = 0.80).

**TABLE 4:** Means, standard deviations, and Pearson zero-order correlations.

	**Variable**	***M***	***SD***	**1**	**2**	**3**	**4**	**5**	**6**	**7**	**8**	**9**	**10**	**11**	**12**
1	Clinical treatment (0 = no, 1 = yes)	0.51	0.50												
2	Sex (0 = boys, 1 = girls)	0.39	0.49	−0.14											
3	Age (years)	4.86	1.32	−0.13	0.11										
4	ICTS frustration	10.02	3.90	0.44^**^	−0.21^**^	−0.02									
5	ICTS inhibition	8.05	3.79	0.09	0.01	−0.05	0.28^**^								
6	ICTS attention	12.23	3.87	−0.60^**^	0.04	−0.06	−0.45^**^	−0.15							
7	ICTS impulsivity	18.80	6.61	0.61^**^	−0.15	0.02	0.85^**^	0.25^**^	−0.85^**^						
8	CBCL aggressive behavior	60.20	10.09	0.31^**^	−0.09	0.13	0.64^**^	0.20^*^	−0.38^**^	0.61^**^					
9	CBCL attention problems	57.46	9.08	0.50^**^	−0.13	0.25^**^	0.53^**^	0.13	−0.67^**^	0.70^**^	0.63^**^				
10	CBCL anxious/depressive	57.04	8.99	0.38^**^	−0.06	0.12	0.46^**^	0.41^**^	−0.44^**^	0.53^**^	0.56^**^	0.56^**^			
11	CBCL withdrawn	58.44	8.05	0.33^**^	−0.15	−0.06	0.39^**^	0.45^**^	−0.31^*^	0.41^**^	0.44^*^	0.42^**^	0.59^**^		
12	CBCL somatic complaints	57.11	8.36	0.37^**^	0.09	0.07	0.37^**^	0.16^*^	−0.37^**^	0.44^**^	0.45^**^	0.44^**^	0.52^**^	0.38^**^	
13	CBCL total problems	58.90	10.89	0.34^**^	−0.11	0.17^*^	0.64^**^	0.34^**^	−0.49^*^	0.67^**^	0.83^**^	0.73^**^	0.77^**^	0.66^**^	0.61^**^

*ICTS, Integrative Child Temperament Screener; CBCL, Child Behavior Checklist. ICTS scores represent total scores, CBCL scores represent T-scores*.

* *p < 0.05*,

** *p < 0.01*.

Table 5 shows the mean level differences in temperament traits between the referred and the non-referred children, along with effect size estimates and AUROC curves for both the ICTS and the ICTI. The AUROC is a measure for the diagnostic efficiency of a measurement. An AUROC of 0.50 indicates that the measurement performs at chance levels, and an AUROC of 1.0 indicates that the measurement performs perfectly. The following AUROC benchmarks have often been used in the literature: 0.90 is “excellent,” 0.80 is “good,” 0.70 is “fair,” and below 0.70 is “poor.” In practice, AUROCs in the range of 0.70 to 0.80 are considered to be realistic of a good test (34). As can be seen from Table 5, ICTS Frustration, ICTS Inattention (reverse-scored ICTS Attention), and the composite variable Impulsivity performed within this range, as did the corresponding ICTI scales. The ICTI scales not included in the ICTS, Activity and Sensory Sensitivity, did not discriminate between the clinically referred and non-referred children.

**TABLE 5:** *t*-Test comparisons and area under the curve of the ICTS and ICTI scales for the referred and non-referred samples.

	**Referred** **(*n* = 87)** ***M* (*SD*)**	**Non-referred** **(*n* = 85)*M* (*SD*)**	***t-*Test**	***p-*Value**	**d** **(95% CI)**	**AUC** **(95% CI)**
**ICTS**						
Frustration	11.68 (3.96)	8.29 (2.98)	6.42	<0.001	0.98 (0.66–1.30)	0.76 (0.68–0.83)
Inhibition	8.37 (3.89)	7.72 (3.68)	1.02	0.31	0.16 (−0.14–0.46)	0.55 (0.46–0.64)
Attention	9.90 (3.68)	14.56 (2.34)	−9.62	<0.001	1.46 (1.12–1.79)	0.84 (0.78–0.90)^a^
Impulsivity	22.70 (6.09)	14.73 (4.16)	9.98	<0.001	1.52 (1.18–1.86)	0.86 (0.81–0.92)
**ICTI**						
Frustration	22.14 (7.41)	16.34 (5.12)	5.92	<0.001	0.91 (0.60–1.23)	0.74 (0.66–0.82)
Inhibition	17.96 (7.05)	16.20 (6.64)	1.68	0.10	−0.25 (−0.05–0.56)	0.57 (0.49–0.66)
Attention	19.88 (6.02)	27.19 (4.68)	−8.85	<0.001	1.36 (1.03–1.69)	0.83 (0.77–0.89)^a^
Activity	23.32 (7.05)	20.39 (6.58)	2.80	<0.01	0.43 (0.13–0.74)	0.62 (0.54–0.71)
Sensory Sensitivity	19.15 (6.80)	16.00 (5.73)	3.28	<0.01	0.51 (0.20–0.81)	0.65 (0.56–0.73)

*ICTS, Integrative Child Temperament Screener; ICTI, Integrative Child Temperament Inventory; CI, confidence interval; d, Cohen's d; AUC, area under the curve. Ranges in parentheses*.

a *AUC value was computed from reverse-scored Attention to make high scores indicate more risk (Inattention)*.

To examine whether the longer and more comprehensive ICTI scales added to the prediction of diagnostic status relative to the shorter and more clinically focused ICTS, we ran a multivariate binary logistic regression. Adding the ICTI scales to the ICTS scales resulted only in marginal incremental utility (Δχ^2^ = 9.7; Δ*R*^2^ = 0.04, *p* = 0.088), suggesting that most of the clinically relevant temperament information is captured by the ICTS scales. Table 6 shows the mean level differences in temperament traits, along with effect size estimates and AUROC curves, for children of both samples combined, who were categorized as being at risk and not at risk for externalizing, internalizing, and total problems according to CBCL categorical scoring guidelines (see Method). The results remained effectively unchanged when age and sex differences between the clinically referred and the general population sample were accounted for (See footnote 1). The results both corroborate and extend those found for prediction of general clinical status. Specifically, externalizing risk status was accurately predicted by ICTS Frustration, Inattention, and Impulsivity. Internalizing risk status was predicted by ICTS Inhibition, though with less accuracy compared with the ICTS predictors for externalizing problems.

**TABLE 6:** *t*-Test comparisons and area under the curve of ICTS scales for children at risk and at no risk for externalizing, internalizing, and total problems according to the CBCL.

**ICTS Scales**	**Externalizing** **(*n* = 42)** ***M* (*SD*)**	**No externalizing^a^** **(*n* = 98)*M* (*SD*)**	***t-*Test**	***p-*Value**	**AUC** **(95% CI)**
Frustration	13.55 (3.31)	8.39 (3.41)	−8.48	<0.001	0.86 (0.79–0.92)
Inhibition	8.62 (4.06)	7.51 (3.70)	−1.58	0.117	0.58 (0.48–0.69)
Inattention^b^	9.69 (4.03)	13.41 (3.14)	5.08	<0.001	0.75 (0.66–0.84)
Impulsivity	24.69 (6.02)	15.98 (5.20)	−8.83	<0.001	0.86 (0.79–0.93)
	**Internalizing** **(*n*** **=** **41)**	**No internalizing** **(*n*** **=** **102)**			
Frustration	12.29 (4.01)	8.52 (3.49)	−5.77	<0.001	0.77 (0.68–0.86)
Inhibition	10.00 (4.10)	7.08 (3.40)	−4.37	<0.001	0.72 (0.62–0.81)
Inttention^b^	10.07 (4.19)	13.41 (3.23)	4.66	<0.001	0.73 (64–0.82)
Impulsivity	23.22 (6.50)	16.04 (5.61)	−6.60	<0.001	0.80 (0.71–0.88)
	**Overall problem behavior** **(*****n*** **=** **51)**	**No overall problem behavior(*****n*** **=** **98)**			
Frustration	12.92 (3.55)	8.22 (3.21)	−8.34	<0.001	0.84 (0.77–0.91)
Inhibition	9.29 (4.00)	7.05 (3.40)	−3.60	<0.001	0.67 (0.58–0.76)
Inattention^a^	9.84 (4.03)	13.76 (3.08)	5.85	<0.001	0.77 (0.69–0.85)
Impulsivity	23.94 (5.76)	15.47 (5.12)	−9.31	<0.001	0.87 (0.81–0.93)

*CBCL, Child Behavior Checklist; ICTS, Integrative Child Temperament Screener; AUC, area under the curve; CI, confidence interval*.

a *Children showing borderline externalizing problems (n = 27), internalizing problems (n = 24), and total problems (n = 18) were excluded from analyses*.

b *Attention was reverse scored and labeled “Inattention” here to make high scores indicate more risk*.

## Prediction of Clinical Status from ICTS and CBCL Scales

Lastly, we conducted a hierarchical binary logistic regression that predicted diagnostic status from CBCL Externalizing and CBCL Internalizing problem scores, gender, and age in a first step, adding the ICTS scales in a second step. As shown in Table 7, the results indicated that, after prediction of diagnostic status by the two broadband CBCL scales, gender and age, the ICTS still added substantially to prediction of diagnostic status, confirming its incremental utility. When using the three specific CBCL subscales that are most closely related to the ICTS scales (aggressive behavior, attention problems, and withdrawn), the amount of incremental variance explained by the ICTS was smaller but still significant (see Table 8). On the whole, the results of the logistic regression converged with the AUROC analyses in showing that anger/frustration and attentional persistence (reverse scored) were the ICTS scales with the best ability to discriminate clinically referred children from general population children.

**TABLE 7:** Summary of a multivariate binary logistic regression analysis predicting clinical treatment from ICTS and CBCL-broadband scales.

	**Model 1**				**Model 2**			
**Predictor**	***OR***	***SE***	***z***	***p***	***OR***	***SE***	***z***	***p***
Constant	0.05	1.21	5.96	0.02	0.08	1.64	2.42	0.12
Age	0.76	0.15	3.57	0.06	0.70	0.19	3.43	0.06
Sex	0.56	0.37	2.48	0.12	0.55	0.50	1.48	0.22
CBCL Externalizing	0.99	0.02	0.15	0.70	0.90	0.03	9.69	<0.01
CBCL Internalizing	1.09	0.02	13.84	<0.01	1.09	0.04	5.96	0.02
ICTS Frustration					1.33	0.09	9.99	<0.01
ICTS Inhibition					1.62	0.07	4.26	0.04
ICTS Inattention^a^					1.59	0.09	28.68	<0.01
Model Summary		χ^2^ = 28.26	*R*^2^ = 0.22	<0.001	χ^2^ = 97.41		*R*^2^ = 0.61	<0.001
Model Comparison (1 vs. 2)		χ^2^ = 69.15	Δ*R^2^ = 0.39*	<0.001				

*N = 160. CBCL, Child Behavior Checklist; ICTS, Integrative Child Temperament Screener*.

a *Attention was reverse-scored and labeled “Inattention” to make high scores indicate more risk*.

**TABLE 8:** Summary of a multivariate binary logistic regression analysis predicting clinical treatment from ICTS and specific CBCL scales.

	**Model 1**				**Model 2**			
**Predictor**	***OR***	***SE***	***z***	***P***	***OR***	***SE***	***z***	***P***
Constant	0.00	2.17	17.62	<0.01	0.00	2.33	10.48	<0.01
Age	0.53	0.22	8.49	<0.01	0.58	0.23	5.90	0.02
Sex	0.70	0.42	0.70	0.40	0.79	0.48	0.25	0.62
CBCL Aggressive Behavior	0.99	0.03	0.10	0.75	0.97	0.04	0.76	0.38
CBCL Withdrawn	1.03	0.03	0.98	0.32	1.06	0.04	2.55	0.11
CBCL Attention Problems	1.22	0.04	23.36	<0.01	1.10	0.05	3.60	0.06
ICTS Frustration					1.18	0.09	3.71	0.05
ICTS Inhibition					0.88	0.08	2.74	0.10
ICTS Inattention^a^					1.45	0.10	14.90	<0.01
Model Summary		χ^2^ = 66.99	*R*^2^ = 0.46	<0.001	χ^2^ = 93.21		*R*^2^ = 0.59	<0.001
Model Comparison (1 vs. 2)		χ^2^ = 26.22	Δ*R^2^ = 0.16*	<0.001				

*N = 160. CBCL, Child Behavior Checklist; ICTS, Integrative Child Temperament Screener*.

a *Attention was reverse-scored and labeled “Inattention” to make high scores indicate more risk*.

## Discussion

### Temperament and Psychopathology

The results of this study extend previous research on temperament and psychopathology by showing that children referred for psychiatric treatment exhibit temperament attributes that are similar to those that have been found to relate to behavior problems in general population studies. In line with previous research that found negative emotionality, particularly anger, in combination with poor self-regulation to have a special role in predicting externalizing disorders (1–3, 5, 17), we found anger-frustration and low attentional persistence to be the factors most distinctively associated with referral status and with an externalizing problem behavior profile in particular. In turn, behavioral inhibition was found to be elevated only in children with an internalizing problem profile. Overall, this pattern suggests that predisposing temperamental factors previously identified in general population samples differ from those present in children referred for psychiatric treatment by degree rather than type.

If children's referral status is taken as a reference, the attributes deviating most strongly from levels found in general population children were low attentional persistence and the composite variable impulsivity. This finding is consistent with CBCL “Attention Problem Scale”-items having shown to have the greatest efficacy in discriminating between referred and non-referred children (35) as well as with the crucial role of low effortful control and low self-control in predicting a broad range of social-emotional problems throughout childhood and up to adulthood (3, 5, 11). That behavioral inhibition did not discriminate between referred and non-referred children may be related to the lower prevalence of internalizing relative to externalizing symptoms in clinically referred preschoolers as well as to the lesser visibility of internalizing compared to externalizing difficulties for parents and professionals in children of this age group (29, 36, 37).

### Screening Utility of the ICTS

Diagnostic status could be well-predicted by the ICTS, thus corroborating its clinical validity and showing promise as a potential screening tool. Somewhat surprisingly, the ICTS predicted children's diagnostic status as accurately as the 100-plus-item CBCL, and even added significantly to the prediction of diagnostic status above and beyond the CBCL. It is also worth noting that clinically relevant temperament information was captured by the nine-item ICTS scales as effectively as by the 30-item ICTI. Three factors may help explain the somewhat unsuspected sensitivity of the ICTS in identifying clinically referred children.

First, a growing body of research points to frustration proneness and low attentional control as core features of the externalizing problem cluster and to behavioral inhibition as a core component of the internalizing cluster. By directly capturing the temperamental core components of externalizing and internalizing psychopathology, the ICTS may achieve a relatively high level of diagnostic accuracy despite its brevity. This notion is supported by the finding that of the 11 CBCL items that have been found to discriminate most powerfully between referred and non-referred children in a large-scale German study (35), many resemble the ICTS items in their focus on deficits in attentional and emotional regulation. Second, the CBCL covers a broad range of problem behaviors, some of which were not exhibited by the clinically referred children. It is possible that the CBCL's diagnostic acuity was weakened by the lack of relevance of some CBCL scales in the present context. This is suggested by the smaller incremental prediction of clinical status by the ICTS over the CBCL, when the CBCL scales that are most closely related to the ICTS were used. A third possible reason is that, because the wording of ICTS items implies less serious behavior problems compared to the wording of many CBCL items, parents might be inclined to answer more truthfully when asked about their children's behaviors. All while providing plausible reasons for the relatively good performance of the ICTS vis-à-vis the CBCL, we should emphasize that the goal of the ICTS is not to serve as a diagnostic tool, but as potential screening device among others. Also, future studies are necessary to determine whether the findings will replicate in other samples.

### Limitations

Results from the current research should be interpreted within its limitations. First, the data was cross-sectional. Therefore, it is difficult to draw conclusions regarding the causal role of the ICTS temperament attributes in the onset of the disorders. Second, although assignment of children to externalizing and internalizing problem status based on the CBCL was consistent with the information used for the ICD-10 classification, and seemed useful in light of the difficulties of ascertaining specific mental disorders during the preschool years (38), the distinction between actual and implied diagnosis needs to be kept in mind in interpreting the CBCL-related findings. Third, and in regard to the prediction of clinical status more generally, it needs to be kept in mind that the clinical significance of the ICTS dimensions was established on the basis of the distribution of disorders in the current clinical sample. Although this distribution seems broadly reflective of the prevalence of preschool disorders as identified in epidemiological studies, additional studies are necessary to particularize the clinal significance of the ICTS scales. Fourth, because of the relatively small sample sizes, it would be premature to draw strong conclusions as to the generalizability of the findings. Finally, the temperament components included in the ICTS were selected on the basis of their early developmental appearance, their predictive validity for behavior disorders over the long term and their measurement-invariant properties. We do not suggest that the ICTS provides an exhaustive assessment of all child temperament dimensions that could potentially place a child at risk for behavior problems. For instance, attentional focusing is a key facet of effortful control that is included in the ICTS (via the attentional persistence scale) because it can be assessed in very young children with items that are both reliable and measurement-invariant over time. However, as children grow older, inhibitory control might also be considered for inclusion in a scale of temperament risk factors.

## Outlook and Conclusions

These limitations notwithstanding, the current research provides one of the first demonstrations that temperament risk factors identified in general population studies are also exhibited by children referred for psychiatric treatment, albeit in more marked form. The finding that these factors can be quickly and accurately assessed by the ICTS substantiates the instrument's clinical validity, thereby commending consideration by child mental health professionals. Indeed, beyond their scientific merits, the current results could also be of value in making prevention and interventions more viable and effective. Specifically, several recent studies have demonstrated the importance of adequate parenting in reducing adverse consequences of child temperament attributes that are similar to those assessed by the ICTS (39–41). Parallel to these research developments, the last decade has also seen the advent of several temperament-based prevention and intervention programs that use parent and teacher guidance (42), behavioral skills training (43), and computer exercises aimed at promoting self-regulation [e.g., (44)] or reducing behavioral inhibition [e.g., (45)].

Promising though these programs are, they will be difficult to put into widespread practice, such as through primary pediatric care or preschool service systems, without a measure that allows for a quick and valid assessment of a child's temperament. In virtue of its brevity and promising screening effectiveness for a relatively broad range of behavioral or emotional problems, the ICTS helps to remove an important barrier to the implementation of programs designed to reduce risks associated with particular temperament attributes.

## Data Availability Statement

The raw data supporting the conclusions of this article will be made available by the authors, without undue reservation.

## Ethics Statement

The studies involving human participants were reviewed and approved by Medical University of Innsbruck Ethics Committee. Written informed consent to participate in this study was provided by the participants' legal guardian/next of kin.

## Author Contributions

MZ developed the research idea. Data collection and processing was done by EM, KS, and CT. MZ, VB, and HS analyzed the data with input from CT. MZ and VB drafted the manuscript with input from all authors. The study design was developed collaboratively by all authors. All authors provided feedback and contributed to critical revisions of the manuscript's draft and approved the final version of the manuscript for submission.

## Conflict of Interest

The authors declare that the research was conducted in the absence of any commercial or financial relationships that could be construed as a potential conflict of interest.

## Footnotes

^1^Because there were some differences in age and sex between the clinical and general population sample (see Table 3), we ran two types of analyses to examine whether these differences could have been a source of bias. First, we re-ran the analyses with a “trimmed” sample, in which children older than 7 years and younger than 3 years were removed. This age-restricted sample (*N* = 69) was characterized by a much lower spread (*SD* = 1.14, down from *SD* = 1.81 in the full sample), all while remaining broadly comparable in regard to the average age (*M* = 4.85 vs. 5.51, for the clinically referred and the general population sample, respectively). Second, we recomputed the analyses controlling for sex and age. Both types of analyses left the results reported in Tables 4–6 effectively unchanged. When changes occurred, they were minimal and did not change the results substantially nor any of our conclusions. The respective analyses are available on request.

## References

[1] Zentner M Shiner R. Fifty years of progress in temperament research: a synthesis of major themes, findings, challenges, and a look forward. In: Zentner M Shiner R, editors. Handbook of Temperament. New York, NY: Guilford Press (2015). p. 673–700.

[2] Lengua L Wachs TD. Temperament and risk: resilient and vulnerable responses to adversity. In: Zentner M Shiner R, editors. Handbook of Temperament. New York, NY: Guilford Press (2015). p. 519–40.

[3] Tackett JL Martel M Kushner S. Temperament, externalizing disorders, and ADHD. In: Zentner M Shiner R, editors. Handbook of Temperament. New York, NY: Guilford Press (2015). p. 562–80.

[4] Klein DN Dyson MW Kujawa AJ Kotov R. Temperament and internalizing disorders. In: Zentner M Shiner R, editors. Handbook of Temperament. New York, NY: Guilford Press (2015). p. 541–61.

[5] DeLisi M Fox BH Fully M Vaughn MG. The effects of temperament, psychopathy, and childhood trauma among delinquent youth: a test of DeLisi and Vaughn's temperament-based theory of crime. Int J Law Psychiatry. (2018) 57:53–60. 10.1016/j.ijlp.2018.01.006 29548504

[6] Scheper FY MajdandŽić M van de Ven PM Jansen LMC Doreleijers TAH Schuengel C. Temperament traits and psychopathology in young clinically referred children compared to a general population sample. Child Psychiatry Hum Dev. (2017) 48:841–50. 10.1007/s10578-016-0708-6 28097446 PMC5680369

[7] Gartstein M Bridgett DJ Low CM. Asking questions about temperament: self and other-report measures. In: Zentner M Shiner R, editors. Handbook of temperament. New York, NY: Guilford Press (2015). p. 183–208.

[8] Buzzell GA Troller-Renfree SV Barker TV Bowman LC Chronis-Tuscano A Henderson HA. A neurobehavioral mechanism linking behaviorally inhibited temperament and later adolescent social anxiety. J Am Acad Child Adolesc Psychiatry. (2017) 56:1097–05. 10.1016/j.jaac.2017.10.007 29173744 PMC5975216

[9] Sandstrom A Uher R Pavlova B. Prospective association between childhood behavioral inhibition and anxiety: a meta-analysis. J Abnorm Child Psychol. (2020) 48:57–66. 10.1007/s10802-019-00588-5 31642030

[10] Liu C Moore GA Beekman C Pérez-Edgar KE Leve LD Shaw DS. Developmental patterns of anger from infancy to middle childhood predict problem behaviors at age 8. Dev Psychol. (2018) 54:2090–100. 10.1037/dev0000589 30265026 PMC6264907

[11] Nigg JT. On the relations among self-regulation, self-control, executive functioning, effortful control, cognitive control, impulsivity, risk-taking, and inhibition for develop-mental psychopathology. J Child Psychol Psychiatry. (2017) 58:361–83. 10.1111/jcpp.12675 28035675 PMC5367959

[12] De Pauw S Mervielde I VanLeeuwen KG. How are traits related to problem behavior in preschoolers? Similarities and contrasts between temperament and personality. J Abnorm Child Psychol. (2009) 37:309–25. 10.1007/s10802-008-9290-0 19165590

[13] Einziger T Levi L Zilberman-Hayun Y Auerbach JG Atzaba-Poria N Arbelle S. Predicting ADHD symptoms in adolescence from early childhood temperament traits. J Abnorm Child Psychol. (2018) 46:265–76. 10.1007/s10802-017-0287-4 28317068

[14] Hill AL Degnan KA Calkins SD Keane SP. Profiles of externalizing behavior problems for boys and girls across preschool: the roles of emotion regulation and inattention. Dev Psychol. (2006) 42:913–28. 10.1037/0012-1649.42.5.913 16953696

[15] Caspi A Silva PA. Temperamental qualities at age three predict personality traits in young adulthood: longitudinal evidence from a birth cohort. Child Dev. (1995) 66:486–98. 10.2307/1131592 7750379

[16] Lee V Duku E Zwaigenbaum L Bennett T Szatmari P Elsabbagh M. Temperament influences the relationship between symptom severity and adaptive functioning in children with autism spectrum disorder. Autism. (2020) 24:2057–70. 10.1177/1362361320933048 32615784

[17] Zentner M. Identifying child temperament risk factors from 2 to 8 years of age: validation of a brief temperament screening tool in the US, Europe, and China. Eur Child Adolesc Psychiatry. (2020) 29:665–78. 10.1007/s00787-019-01379-5 31414220 PMC7250798

[18] Zentner M Wang F. ICTI: Integrative Child Temperament Inventory Manual. Oxford: Hogrefe (2013).

[19] McDevitt SC Carey WB. The measurement of temperament in 3–7 year old children. J Child Psychol Psychiatr. (1978) 19:245–53. 10.1111/j.1469-7610.1978.tb00467.x 681467

[20] Rothbart MK Ahadi SA Hershey KL Fisher P. Investigations of temperament at 3-7 years: the Children's Behavior Questionnaire. Child Dev. (2001) 72:1394–408. 10.1111/1467-8624.00355 11699677

[21] Buss AH Plomin R. Temperament: Early Developing Personality Traits. Hillsdale: Erlbaum (1984).

[22] Bates JE Freeland CA Lounsbury ML. Measurement of infant difficultness. Child Dev. (1979) 1:794–803. 10.2307/1128946 498854

[23] Constantino JN Cloninger CR Clarke AR Hashemi B Przybeck T. Application of the seven-factor model of personality to early childhood. Psychiatry Res. (2002) 109:229–43. 10.1016/S0165-1781(02)00008-2 11959360

[24] Goldsmith HH. Studying temperament via construction of the Toddler Behavior Assessment Questionnaire. Child Dev. (1996) 67:218–35. 10.2307/1131697 8605830

[25] de la Osa N Granero R Penelo E Domènech JM Ezpeleta L. The short and very short forms of the Children's Behavior Questionnaire in a community sample of preschoolers. Assessment. (2014) 21:463–76. 10.1177/1073191113508809 24235176

[26] Faul F Erdfelder E Buchner A Lang AG. Statistical power analyses using G^*^ Power 3.1: Tests for correlation and regression analyses. Behav Res Methods. (2009) 41:1149–60. 10.3758/BRM.41.4.1149 19897823

[27] Schneider S Pflug V In-Albon T Margraf J. Kinder-DIPS Open Access: Diagnostisches Interview bei psychischen Störungen im Kindes- und Jugendalter. Bochum: Forschungs- und Behandlungszentrum für psychische Gesundheit, Ruhr-Universität Bochum (2017).

[28] Egger HL Angold A. Common emotional and behavioral disorders in preschool children: presentation, nosology, and epidemiology. J Child Psychol Psychiatry. (2006) 47:313–37. 10.1111/j.1469-7610.2006.01618.x 16492262

[29] Wichstrøm L Berg-Nielsen TS Angold A Egger HL Solheim E Sveen TH. Prevalence of psychiatric disorders in preschoolers. J Child Psychol Psychiatry. (2012) 53:695–705. 10.1111/j.1469-7610.2011.02514.x 22211517

[30] Steffen A Akmatov MK Holstiege J Bätzing J. Diagnoseprävalenz psychischer Störungen bei Kindern und Jugendlichen in Deutschland: eine Analyse bundesweiter vertragsärztlicher Abrechnungsdaten der Jahre 2009 bis 2017. Berlin (2018). Available online at: https://www.versorgungsatlas.de/fileadmin/ziva_docs/93/VA_18-07_Bericht_PsychStoerungenKinderJugendl_V2_2019-01-15.pdf (accessed May 13, 2021).

[31] Achenbach TM Rescorla LA. Arbeitsgruppe Deutsche Child Behavior Checklist. CBCL 11/2−5. Deutschsprachige Fassung der Child Behavior Checklist for Ages 11/2−5 von T.M. Achenbach. Göttingen: Hogrefe (2014).

[32] Döpfner M Plück J Kinnen C. CBCL/6–18R, TRF/6–18R, YSR/11–18R. Deutsche Schulalter-Formen der Child Behavior Checklist von Thomas M. Achenbach. Göttingen: Hogrefe (2014).

[33] Döpfner M Schmeck K Berner W. Handbuch: Elternfragebogen über das Verhalten von Kindern und Jugendlichen. Forschungsergebnisse zur deutschen Fassung der Child Behavior Checklist (CBCL). Köln: Arbeitsgruppe Kinder-, Jugend- und Familiendiagnostik (1994).

[34] Rice ME Harris GT. Comparing effect sizes in follow-up studies: ROC area, Cohen's d, and r. Law Hum Behav. (2005) 29:615–20. 10.1007/s10979-005-6832-7 16254746

[35] Schmeck K Poustka F Döpfner M Pluck J Berner W Lehmkuhl G. Discriminant validity of the child behaviour checklist CBCL-4/18 in German samples. Eur Child Adolesc Psychiatry. (2001) 10:240–7. 10.1007/s007870170013 11794549

[36] Achenbach TM Howell CT Quay HC Conners CK Bates JE. National survey of problems and competencies among four-to sixteen-year-olds: Parents' reports for normative and clinical samples. Monogr Soc Res Child Dev. (1991) 56:i−130. 10.2307/1166156 1770964

[37] Gilliom M Shaw S. Codevelopment of externalizing and internalizing problems in early childhood. Dev Psychopathol. (2004) 16:313–33. 10.1017/S0954579404044530 15487598

[38] Bolten M. Clinical diagnostics in preschool-aged children (0 – 6 years): Challenges and praxis parameters. Kindh Entwickl. (2020) 29:178–92. 10.1026/0942-5403/a000316

[39] Perra O Paine AL Hay DF. Continuity and change in anger and aggressiveness from infancy to childhood: the protective effects of positive parenting. Dev Psychopathol. (2020). 10.1017/S0954579420000243. [Epub ahead of print]. 32635948

[40] Smith CL Day KL. Parenting, anger, and effortful control as predictors of child externalizing behavior: the role of child sex as a moderator. Int J Behav Dev. (2018) 42:248–56. 10.1177/0165025417692898

[41] Weeland J Chhangur RR Jaffee SR Van Der Giessen D Matthys W De Castro BO. Does the Incredible Years reduce child externalizing problems through improved parenting? The role of child negative affectivity and serotonin transporter linked polymorphic region (5-HTTLPR) genotype. Dev Psychopathol. (2018) 30:93–112. 10.1017/S0954579417000499 28434415

[42] McClowry SG Collins A. Temperament-based intervention: reconceptualized from a response to intervention framework. In: Zentner M Shiner R, editors. Handbook of Temperament. New York, NY: Guilford Press (2015). p. 607–27.

[43] Lau EX Rapee RM Coplan RJ. Combining child social skills training with a parent early intervention program for inhibited preschool children. J Anxiety Disord. (2017) 51:32–8. 10.1016/j.janxdis.2017.08.007 28910693

[44] Rueda C. Effortful control. In: Zentner M Shiner R, editors. Handbook of Temperament. New York, NY: Guilford Press. (2015). p. 145–67.

[45] Liu P Taber-Thomas BC Fu X Pérez-Edgar KE. Biobehavioral markers of attention bias modification in temperamental risk for anxiety: a randomized control trial. J Am Acad Child Adolesc Psychiatry. (2018) 57:103–10. 10.1016/j.jaac.2017.11.016 29413142 PMC6409187

