# Supplementary material for: Early Detection of Temperament Risk Factors: A Comparison of Clinically Referred and General Population Children
Source: Front Psychiatry. 2021 Jun 24;12:667503. doi: 10.3389/fpsyt.2021.667503 (PMC8264422; doi:10.3389/fpsyt.2021.667503)
Supplement: Supplementary file 1 [file Table_1.DOCX]

Supplementary Table 1. Frequencies of ICD-10 diagnoses in the subsample of clinically referred children (n = 87).

| **ICD-10** | **Description** | **n** | **%** |  | **ICD-10** | **Description** | **n** | **%** |
| --- | --- | --- | --- | --- | --- | --- | --- | --- |
|  | No diagnosis | 20 | 23.0 |  | - | No Axis 1 diagnosis | 19 | 21.8 |
|  |  |  |  |  | - | Healthy child | 1 | 1.1 |
| F40-F48 | Neurotic, stress-related and somatoform disorders | 9 | 10.3 |  | F43 | Reaction to severe stress, and adjustment disorders | 9 | 10.3 |
| F50-F59 | Behavioral syndromes associated with physiological disturbances and physical factors | 7 | 8.0 |  | F50 | Eating disorders | 2 | 2.3 |
|  |  |  |  |  | F51 | Non-organic sleep disorders | 5 | 5.7 |
| F80-F89 | Disorders of psychological development | 8 | 9.2 |  | F80 | Specific developmental disorders of speech and language | 1 | 1.1 |
|  |  |  |  |  | F84 | Pervasive developmental disorders | 6 | 6.9 |
|  |  |  |  |  | F89 | Unspecified disorder of psychological development | 1 | 1.1 |
| F90-F98 | Behavioral and emotional disorders with onset usually occurring in childhood and adolescence | 54 | 62.1 |  | F90 | Attention-deficit hyperactivity disorders | 2 | 2.3 |
|  |  |  |  |  | F92 | Mixed disorders of conduct and emotions | 2 | 2.3 |
|  |  |  |  |  | F93 | Emotional disorders with onset specific to childhood | 9 | 10.3 |
|  |  |  |  |  | F94 | Disorders of social functioning with onset specific to childhood and adolescence | 2 | 2.3 |
|  |  |  |  |  | F95 | Tic disorder | 1 | 1.1 |
|  |  |  |  |  | F98 | Other behavioral and emotional disorders with onset usually occurring in childhood and adolescence | 42 | 48.3 |
|  |  |  |  |  |  |  |  |  |
